# A causal model of critical thinking in a sample of Iranian medical students: associations with self-esteem, hardiness, and positive affect

**DOI:** 10.3205/zma001251

**Published:** 2019-08-15

**Authors:** Mahbobeh Faramarzi, Soraya Khafri

**Affiliations:** 1 Social Determinants of Health Research Center, Health Research Institute, Babol University of Medical Sciences, Babol, Iran; 2 Infertility and Reproductive Health Research Center, Health Research Institute, Babol University of Medical Sciences, Babol, Iran

**Keywords:** critical thinking, self-esteem, affect

## Abstract

**Background: **Medical students’ ability to think critically influences professional decision-making processes and may have direct and indirect implications for the quality of medical care. Few studies have previously investigated the role of psychological factors in the prediction of critical thinking among university students. The current study addresses the testing a model that examines the effect of self-esteem, psychological hardiness (the positive capacity to cope with stress) and positive affect (how much emotions are experienced as positive affects) on critical thinking.

**Methods: **In a cross-sectional study, 200 medical sciences students enrolled at the Babol University of Medical Sciences (Babol City, Iran) were randomly invited to enter the study during the 2014/15 academic year. The participants completed four reliable and valid questionnaires: California Critical Thinking Skills Test Form B (CCTST-B); Positive Affect Schedule (PAS); Ahvaz Psychological Hardiness Inventory (AHI); and Rosenberg Self-Esteem Scale (RSES). A causal model of the relationship between variables was tested using path analysis. We used the software Statistical Package for Social Sciences (SPSS) v.23 for the regression model to test the suitable models and fitness of the components. P<0.05 was considered significant.

**Results: **Self-esteem had a significant positive direct effect on critical thinking (β=0.458). The number of semesters the students had passed had a significant direct effect on critical thinking (β=0.249). Neither psychological hardiness nor positive affect had direct effects on the students’ critical thinking. An indirect positive mediating effect was revealed between psychological hardiness and critical thinking through self-esteem (β=0.177). Also, positive affect had an indirect significant effect on critical thinking through self-esteem (β=0.189).

**Conclusion: **Self-esteem mediates the effect of psychological hardiness and positive affect on critical thinking in medical students.

## Zusammenfassung

**Hintergrund: **Die Fähigkeit von Studierenden Medizin, kritisch zu denken, beeinflusst professionelle Entscheidungsprozesse und hat möglicherweise direkte und indirekte Auswirkungen auf die Qualität der medizinischen Versorgung.Wenige Studien haben zuvor die Rolle von psychologischen Faktoren für die Vorhersage des kritischen Denkens bei Universitätsstudenten untersucht. Die aktuelle Studie überprüft ein Modell, das die Wirkung von Selbstwertgefühl, psychologischer Widerstandsfähigkeit (die positive Fähigkeit, mit Stress umzugehen) und positivem Affekt (welcher Anteil an Emotionen als positive Affekte erfahren werden) auf kritisches Denken darstellt.

**Methoden:** In einer Querschnittstudie wurden 200 Studierende der Medizin, die an an der Babol Universität der Medizinischen Wissenschaften (Babol-Stadt, Iran) eingeschrieben waren, randomisier eingeladen, an der Studie während des Studienjahres 2014/15 teilzunehmen. Die Teilnehmer füllten vier reliable und valide Fragebögen aus: California Critical Thinking Skills Test Form B (CCTST-B); Positive Affect Schedule (PAS); Ahvaz Psychological Hardiness Inventory (AHI); Rosenberg Self-Esteem Scale (RSES). Ein Kausalmodell der Beziehung zwischen Variablen wurde mittels Pfadanalyse getestet. Wir benutzten SPSS v.23 für das Regressionsmodell, um die geeigneten Modelle und die Fitness der Komponenten zu testen. P<0,05 wurde als signifikant angesehen.

**Ergebnisse: **Das Selbstwertgefühl hatte eine signifikante positive direkte Wirkung auf das kritische Denken (β=0.458). Die Anzahl der Semester, die die Studierenden bestanden hatten, hatte einen bedeutenden direkten Einfluss auf das kritische Denken (β=0,249). Weder die psychologische Widerstandsfähigkeit noch der positive Affekt hatten direkte Auswirkungen auf das kritischeDenken der Studenten..Ein indirekter positiver Mediatoreffekt wurde zwischen psychologischer Widerstandsfähigkeit und kritischem Denken durch das Selbstwertgefühl vermittelt (β=0.177). Auch positiver Affekt hatte einen indirekten, signifikanten Einfluss auf das kritische Denken vermittelt durch das Selbstwertgefühl (β=0,189).

**Schlussfolgerung: **Das Selbstwertgefühl vermittelt den Effekt der psychologischen Widerstandsfähigkeit und positiven Affekt auf das kritische Denken bei Medizinstudenten.

## Background

Critical thinking is defined as “disciplined, self-directed thinking that exemplifies the perfections of thinking appropriate to a particular mode or domain of thought” [1]. Critical thinking includes evaluation, inference, analysis, deductive and inductive reasoning [2]. Critical thinking relates to a high level of cognitive thinking including analysis, synthesis and evaluation [3]. Medical sciences students consistently encounter many new problems in clinical settings and in their medical education [4], [5], and critical thinking is an essential skill in developing a better approach to problem-solving [6]. Previous studies have mentioned critical thinking as a necessary skill for medical students and professionals [7]. 

Critical thinking influences professional decision-making processes [8] and may have an effect on the quality of medical care [9]. Medicine is characterized by the need for inferences, interpretation, intellectual reasoning, and creativity. Therefore, the abilities to find the best solutions, adapt to new situations and make novel decisions are important for medical students [10]. There is a positive relationship between critical thinking attitudes and students’ academic achievement [11]. Critical thinking skills predict academic success throughout the preclinical years of medical education [12].

Many different environmental and personal factors affect the development of critical thinking skills. A meta-analysis found that the factors related to critical thinking skills are teaching factors, student factors, personal factors and child rearing/training [13]. Personal characteristics like motivational beliefs and self-esteem are related to the development of critical thinking [14]. A recent meta-analysis of factors related to critical thinking abilities has reported on the important roles of motivational factors, emotional quotient, and individual and caring factors [15].

According to psychological perspectives, critical thinking requires the ability of mental dispositions to evaluate specific beliefs, claims and actions. Critical thinking relates to both cognitive abilities and affective dispositions. Disposition is defined in terms of critical thinking as the “consistent internal motivation to use critical thinking skills to decide what to believe and what to do” when one approaches problems, ideas, decisions or issues. While critical thinking is typically associated with cognitive abilities and skills, the ideal critical thinker is also characterized by how she or he approaches a specific problem or life in general [16]. Critical thinking skills and dispositions are considered to be highly related. Students equipped with more positive dispositions tend to demonstrate better critical thinking skills, whereas students with negative dispositions tend to exhibit poorer critical thinking skills [16]. Therefore, students with strong critical thinking dispositions tend to have more positive attitudes toward their own critical thinking abilities and are open to unfamiliar situations and ideas [17].

**Figure 1:**
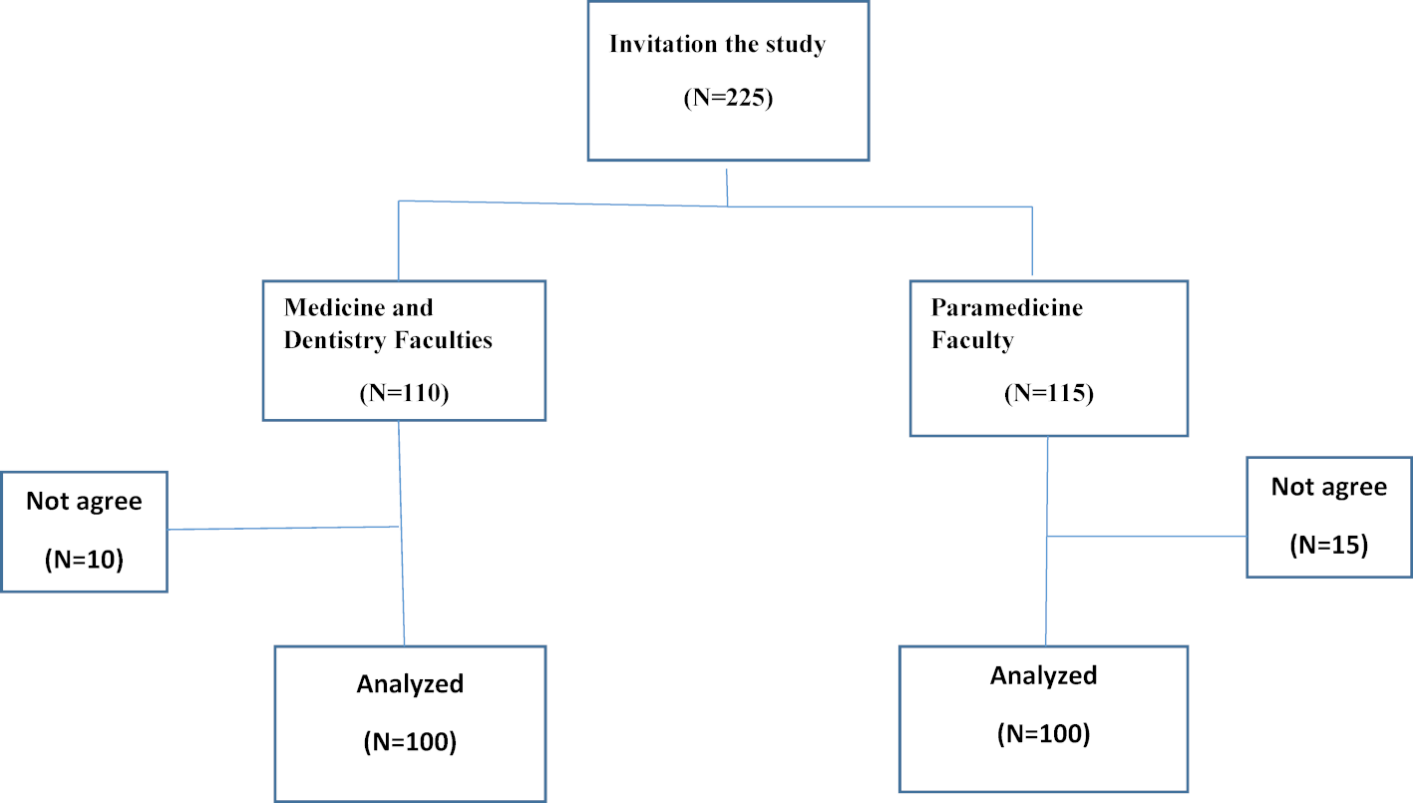
Flow chart of subjects during the study

Some previous studies have emphasized the role of psychological factors in students’ critical thinking [13], [14], [15], [16], [17]. However, there is not enough information on possible links between psychological factors and critical thinking. Little is known about the structural equations through which psychological factors influence critical thinking. The current study addresses the existing gap in research concerning the interaction between psychological factors and critical thinking by testing a model that examines the effect of self-esteem, psychological hardiness and positive affect on critical thinking. Psychological hardiness is a conceptual framework for the positive capacity to cope with stress. Positive affect refers to a human characteristic to describe how much emotions are experienced in positive senses. To the authors’ knowledge, this is the first study using path analysis to examine the impact of self-esteem, psychological hardiness and positive affect on critical thinking in medical students. We investigated whether positive affect, self-esteem and psychological hardiness are related to critical thinking. Also, we examined whether the effect of psychological hardiness and positive affect on critical thinking in medical students was mediated by self-esteem. The aims of the study were to:

1. explore whether psychological hardiness and positive affect are associated with critical thinking;
2. explore whether self-esteem is associated with critical thinking;
3. explore the association between psychological hardiness, positive affect and self-esteem with critical thinking;
4. test the model depicted in figure 1 (Fig. 1) proposing interrelationships between psychological hardiness, positive affect and self-esteem with critical thinking.

## Method

This paper is part of a large study investigating the psychological profile of medical sciences students planned by the Social Determinants of Health Research Center of Babol University. The Medical Ethics Committee at Babol University approved the study (grant no. 9237222). Mental profiles of the students have been reported previously [18]. This paper focuses on critical thinking. A correlational cross-sectional study was utilized to answer the exploratory questions. The target population of the present study was 200 students at the three faculties (Medicine, Dentistry and Paramedicine) of the Babol University of Medical Sciences who were enrolled during the 2014/15 academic year. The Medical and dental students were studying at the doctoral level; paramedicine students at the bachelor level. The required example measure was 193 people, with a base connection between critical thinking and psychological variables of 0.2, significant level of 5% and intensity of 80%, utilizing G Power 3.0.1. Stratified multistage random inspection was utilized to enlist the subjects. In the first stage, schools were considered as strata. Out of seven schools at the Babol University of Medical Sciences, three faculties (Medicine, Dentistry and Paramedicine) were chosen with straightforward arbitrary inspecting amid the 2014/15 academic year. In the second stage, classes at every faculty were considered as strata. Five classes of medicine and six classes of dentistry and twenty classes of paramedicine were chosen by basic random examining. Exclusion criteria extended to the students who were taking the first semester of their first year. Since these students had not yet completed any university coursework, they did not have a grade point average (GPA). In the third stage, 9-10 understudies chose basic irregular inspection per class. Finally, 110 medical and dental students and 115 paramedical students were asked to fill out the questionnaires. Of these, 200 completed the surveys and were included in the final analysis. Figure 1 (Fig. 1) shows the flowchart of subject sampling design. 

All participants completed the four questionnaires and provided demographic data. The questionnaires used were the California Critical Thinking Skills Test Form B (CCTST-B), the Positive Affect Schedule (PAS), the Ahvaz Psychological Hardiness Inventory (AHI) and the Rosenberg Self-Esteem Scale (RSES). Approval for this study was obtained from the Medical Ethics Committee at the Babol University of Medical Sciences. The Persian version of the four instruments was used.

### Assessments

#### California Critical Thinking Skills Test Form B (CCTST-B)

This questionnaire is designed to assess students' critical thinking skills. **CCTST-B** assesses cognitive skills in five areas: evaluation, inference, analysis, deductive reasoning and inductive reasoning. It has 34 multi-optional items with only one true answer. The total score ranges from 0-34. The higher scores indicate strong critical thinking. The Persian version of the CCTST-B was used in this study. The Persian CCTST-B reported that the confidence coefficient of the scale was 0.62 and the construct validity of all subscales was between 0.60–0.65, with a high positive correlation [19]. The students had 45 minutes to complete the questionnaire. 

##### Positive Affect Schedule (PAS)

Positive affect is one aspect of positive emotions related to mood states accompanied by positive feelings, thoughts and behaviors [20]. We used the 10-item mood scale for negative affect (PA) of the PANAS (Positive Affect Negative Affect Schedule). The PANAS developed by Watson et al. (1988) consists of two 10-item mood scales (positive affect and negative affect). They reported that the scales were shown to have high internal consistency and to be stable at appropriate levels over a two-month time period. Subjects were asked to rate the extent to which they experienced particular positive emotions during the past week. The five-point scale has a range of 1 (very slightly or not at all), 2 (a little), 3 (moderately), 4 (quite a bit), and 5 (very much). High positive affect represents an absence of feelings of distress [20]. We used a valid Persian version of the PA-PANAS [21]. The students had 10 minutes to complete the questionnaire. 

##### Ahvaz Psychological Hardiness Inventory (AHI)

This instrument consists of 27 items which are rated on a four-point Likert scale ranging from strongly disagree (0) to strongly agree (3). Total score range is 0–81. Achieving a higher score on this scale indicates psychological hardiness in that person. AHI has good levels of reliability and validity. The validity was reported at 0.83 using Cronbach’s alpha [22]. The students had 30 minutes to complete the questionnaire. 

##### Rosenberg Self-Esteem Scale (RSES)

Developed by Rosenberg (1965), the RSES consists of 10 items which are rated on a four-point Likert scale ranging from strongly agree to strongly disagree [23]. The Persian version of the RSES has good levels of reliability and validity [24]. The students had 10 minutes to complete the questionnaire.

#### Statistical analysis

The descriptive characteristics of the participants are explained as percentages for the categorical variables and mean and standard deviation for the continuous variables. The chi-square test was used to test the relation between two categorical variables. Pearson coefficients were applied to assess the relationship between variables. The t-test was used to determine the differences between the means for men and women. We used path analysis to calculate the direct and indirect effects of independent variables on the dependent variables. At first, we developed a causal model assumption based on previous studies [11], [12], [13], [14], [15], [16], [17], [18]. Then, we tested the hypothetical model with multiple regression analysis. Covariates were removed from the final model if they were not significant (P≥0.05) in multiple regression models. The standard beta coefficient (β) obtained from the multiple regression model represented a correlation coefficient between variables and R^2^ (coefficient of determination). We used the software Statistical Package of Social Sciences (SPSS) v.23 for the regression model to test the suitable models and fitness of the components. P<0.05 was considered significant.

## Results

### Description of study population and relationships

Table 1 (Tab. 1) shows the demographic characteristics of the medical sciences students based on gender. Only the percentage of passed semesters and educational degree level between men and women were statistically significant (*P*=0.002 and P<0.001, respectively).

Table 2 (Tab. 2) shows the mean and standard deviation of psychological hardiness, positive affect, self-esteem and critical thinking, based on gender. The means for positive affect, self-esteem, psychological hardiness, inference, deductive reasoning, inductive reasoning, and total critical thinking were significantly higher for women than for men (P<0.05).

Table 1 (Tab. 1) shows that the population of women and men differed in number of passed semesters. While table 2 (Tab. 2) shows that the critical thinking of men and women varied, the factor influencing the difference in the critical thinking in men and women was the number of passed semesters, not gender itself. For this reason, the number of passed semesters was entered in the final path model of the analysis, not gender.

The associations between psychological hardiness, positive affect, self-esteem and critical thinking are shown in table 3 (Tab. 3). There was a positive significant relationship between critical thinking and psychological hardiness, positive affect and self-esteem (*P*<0.05).

#### Testing the psychological model of critical thinking

**Figure 2:**
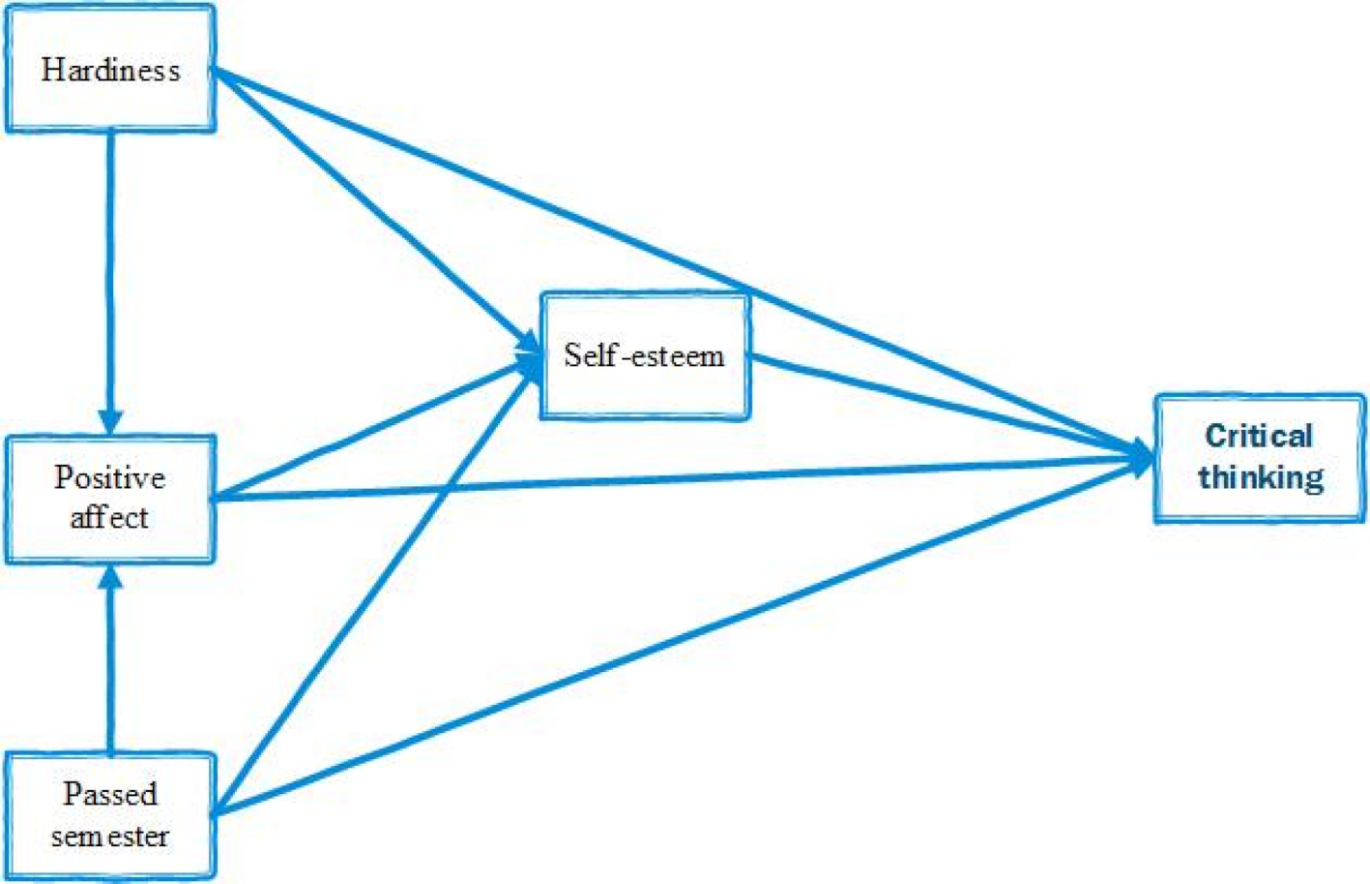
Conceptual model hypothesizing psychological hardiness, positive effect, self-esteem, number of passed semesters, and critical thinking

The theoretical model of the relationship between the variables is shown in figure 2 (Fig. 2). We hypothesized that psychological hardiness, positive affect, self-esteem, and the number of semesters passed by the students had direct effect on the students’ critical thinking. Furthermore, the effect of psychological hardiness, positive affect, self-esteem, and number of passed semesters was mediated by self-esteem (see figure 2 (Fig. 2)).

**Figure 3:**
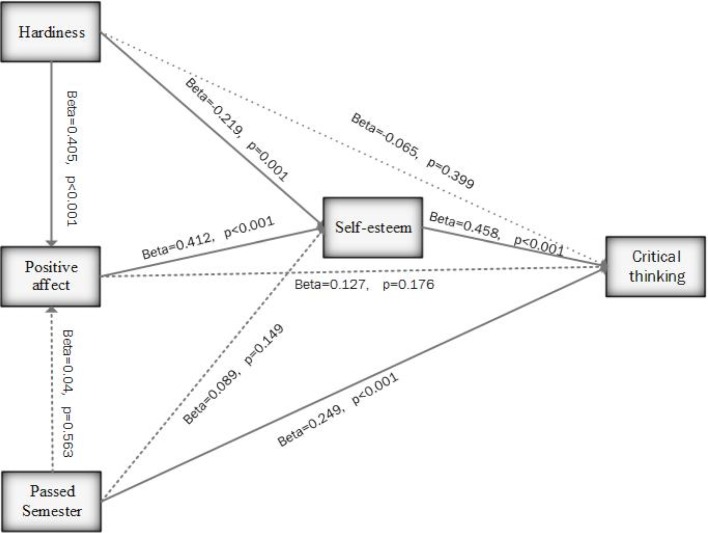
Final path model with standardized path coefficients of the effect of hardiness, positive effect, and passed semester on critical thinking of medical students mediated by self-esteem

Figure 3 (Fig. 3) shows the final model, presenting the significant relationships between psychological hardiness, positive affect and self-esteem with critical thinking in the students using the Pearson correlation coefficient. The significant pathways leading to critical thinking in the model accounted for 29% of the variance in self-esteem and 16.4% of the variance in positive affect. Overall, 30.3% of the changes of the independent variables in this study were explained by critical thinking. Direct effect was not revealed between psychological hardiness and critical thinking; however, an indirect mediating effect was revealed between psychological hardiness and critical thinking through self-esteem (*P*<0.05). The second important variable was positive affect; the results showed that it had no direct effect on critical thinking (*P*>0.05). An indirect mediating effect was revealed in the association between and positive affect and critical thinking through self-esteem (*P*<0.05). Also, direct effect was revealed between the number of semesters the students had passed and critical thinking. 

#### Direct and indirect effects

Table 4 (Tab. 4) shows direct, indirect and total effect calculated by path analysis. The number of semesters passed by the students had a significant direct effect on critical thinking (β=0.249). An indirect positive mediating effect was revealed between psychological hardiness and critical thinking through self-esteem (β=0.177). In addition, positive affect had an indirect significant effect on critical thinking through self-esteem (β=0.189). Self-esteem had a significant positive direct effect on critical thinking (β=0.458).

## Discussion

### Description of study population and relationships

Demographic characteristics showed that women had completed more semesters than men at the doctoral level (*P*<0.05). Our study showed that women had a higher mean for positive affect, self-esteem, psychological hardiness, inference, deductive reasoning, inductive reasoning, and total critical thinking than men did. It seems that the mean differences between men and women for positive affect, self-esteem, psychological hardiness, inference, deductive reasoning, inductive reasoning, and total critical thinking may be related to the women’s higher level of education compared to the men, and are not related to gender differences.

#### Testing the psychological model of critical thinking

The strong positive relationship paths between psychological hardiness and critical thinking were found indirectly through self-esteem. Psychological hardiness as an important trait seems to influence a person’s performance in stressful conditions. People with a high degree of psychological hardiness have three general characteristics: control, commitment and challenge. Hardy people believe that they have control over events, are committed and perceive changing environments as challenging and an opportunity for growth [25]. Few published studies have reported on the relationship between psychological hardiness and self-esteem, and no study has previously reported on the effect of psychological hardiness on critical thinking. There is a link between psychological hardiness and psychological well-being [26]. Psychological hardiness is an important factor in buffering the effects of stress [27]. A study showed that the relationships between psychological hardiness and responses to a specific stressful situation are mediated by coping style and coping self-efficacy [28]. Shekarey et al. (2010) reported a direct and significant correlation between self-efficacy and psychological hardiness. Moreover, they confirmed that self-efficacy and psychological hardiness have important roles in the educational progression of the students [29]. A study showed that there is significant correlation between psychosocial psychological hardiness and thinking styles [18]. How does psychological hardiness contribute to reducing the amount of psychological distress in events? There are some mechanisms to explain this. First, psychological hardiness reduces the perceived threat and increases the person’s expectation that coping efforts will be successful [30]. Second, psychological hardiness is associated with the individual’s use of problem-solving focused on coping strategies dealing with stressful situations [31].

#### Direct and indirect effect

Our data support the conclusion that the main factor contributing to critical thinking is self-esteem. Self-esteem reflects "a person's overall subjective emotional evaluation of his or her own worth" [32]. Few previous studies have been published assessing the effects of self-esteem on critical thinking. Suliman and Halabi (2007) reported that critical thinking was positively correlated with self-esteem [33]. Barkhordary et al. [2009] conducted a study on 170 third- and fourth-year nursing students in Yazd and concluded that there was a significant relationship between critical thinking and self-esteem [34]. Pilevarzadeh et al. (2014) showed that students with higher self-esteem have more favorable critical thinking [35]. How did self-esteem contribute to improving critical thinking? There are some reasons to explain this. First, self-esteem influences all levels of a person’s life, including their thinking, feeling and actions [36]. Second, self-esteem and having independence, confidence and responsibility are essential for medical students to engage in a proper decision–making process and make judgments in different clinical situations [37] .

Our results revealed an indirect positive mediating effect between positive affect and students' critical thinking through self-esteem. Few published studies have previously reported a relationship between critical thinking and affect. Suliman and Halabi (2007) reported that critical thinking was negatively correlated with state anxiety [33]. Esmaeili and Bagheri (2015) found significant correlation between critical thinking and affective control in students [38].

## Limitations

This study has some limitations. First, we used self-reported measures and not performance measures. Second, the present results were gathered at the Medical Sciences University, so they should be generalized with caution to other universities. Third, our sample had high levels of attrition. Approximately 10% of the students who were asked to participate in the study refused to participate, causing selection bias to threaten the results. The question arose as to whether this sample was representative of the population of medical students.

These findings underline the importance of self-esteem mediating processes that explain how psychological hardiness and positive affect produce their effects on critical thinking. Moreover, investigating the association between critical thinking and psychological factors in clinical medical students is recommended. Also, more research is necessary to determine the effect of psychological factors on critical thinking regarding the performance of medical students. This study also suggests the need to explore other variables: how might the interaction of psychological hardiness, self-esteem, positive affect and critical thinking influence clinical judgment in medical students? Could enhancing the levels of psychological hardiness, self-esteem and positive affect improve critical thinking in medical students? These questions need to be addressed in further research.

## Conclusion

The findings suggest that the main factor contributing to critical thinking is self-esteem. In addition, the association between psychological hardiness and positive affect and critical thinking was mediated through self-esteem.

## Funding

This study was supported by a grant (no. 9237222) from a research project at the Babol University of Medical Sciences. We wish to acknowledge all subjects who participated in this study.This study was supported by a grant (no. 9237222) from a research project at the Babol University of Medical Sciences. We wish to acknowledge all subjects who participated in this study.

## Competing interests

The authors declare that they have no competing interests. 

## Figures and Tables

**Table 1:**
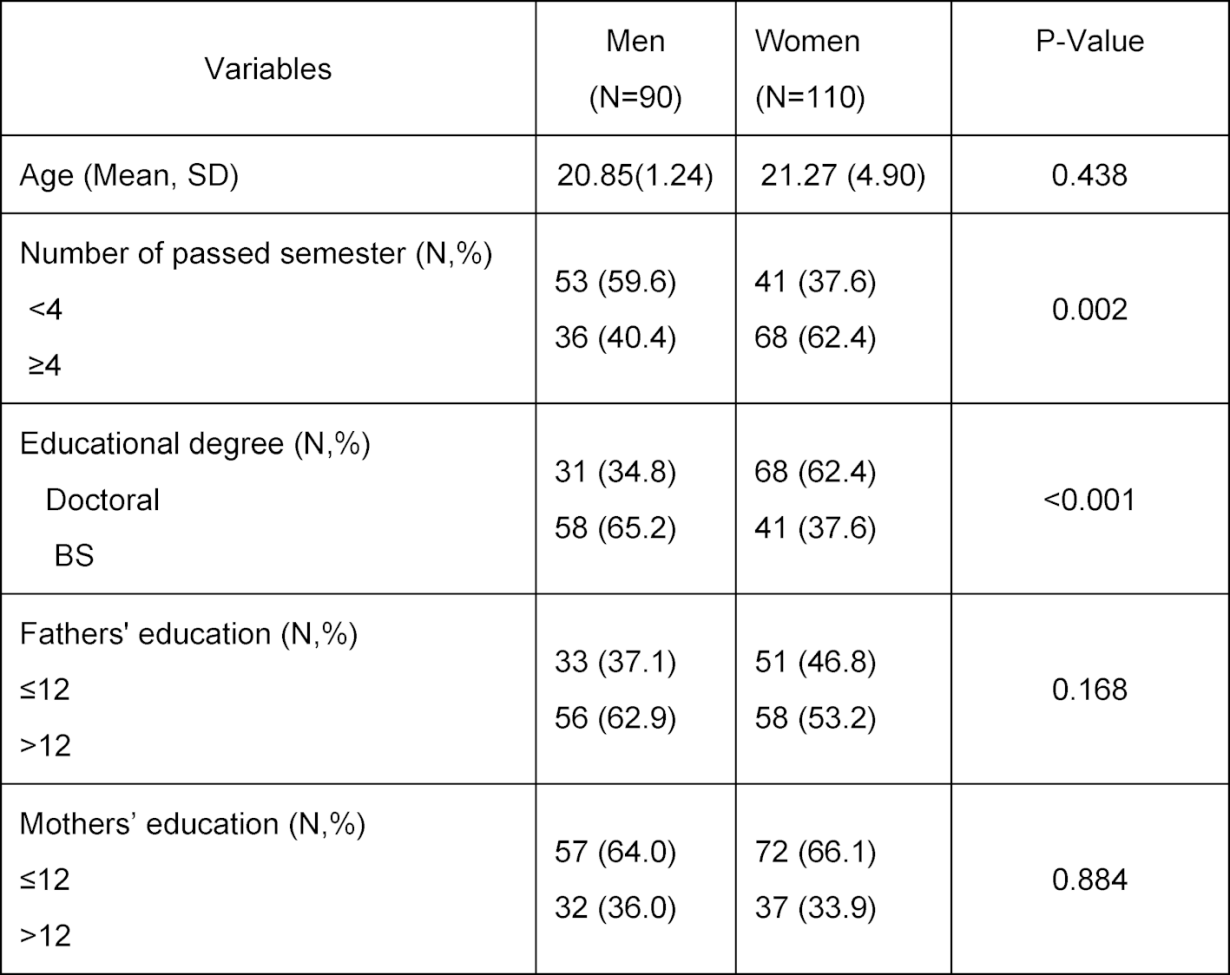
Demographic characteristics of the sample

**Table 2:**
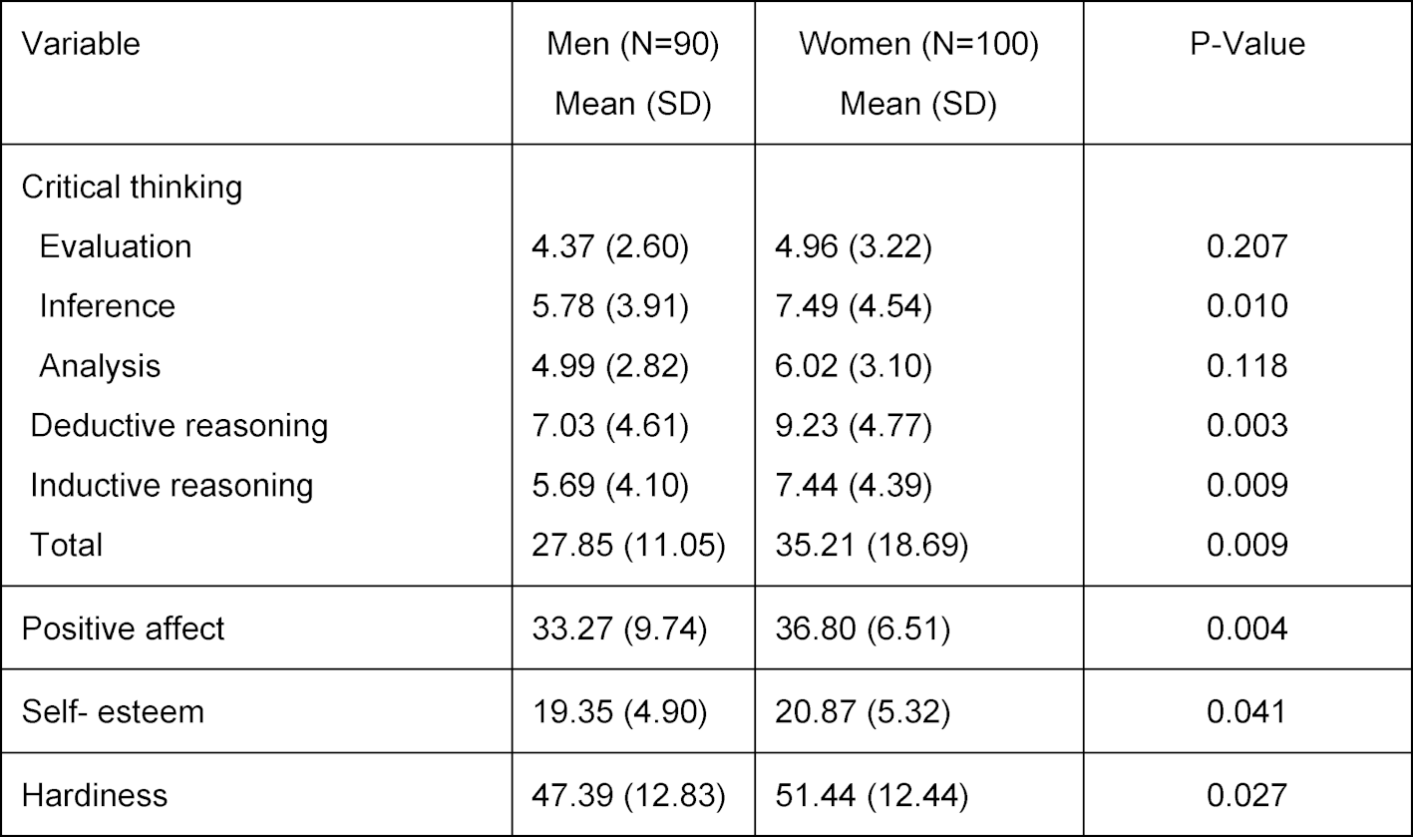
Descriptive indexes of model variables

**Table 3:**
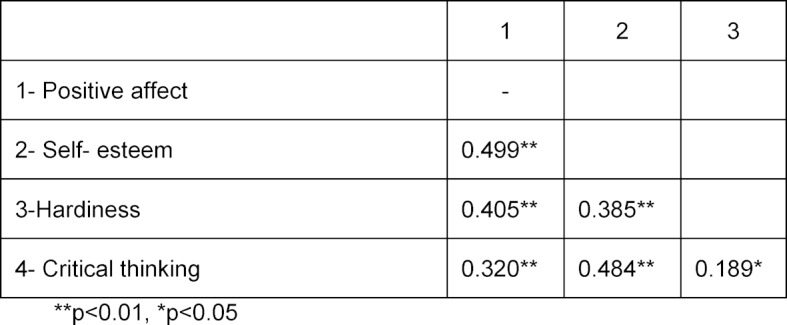
Correlation coefficient between models variables

**Table 4:**
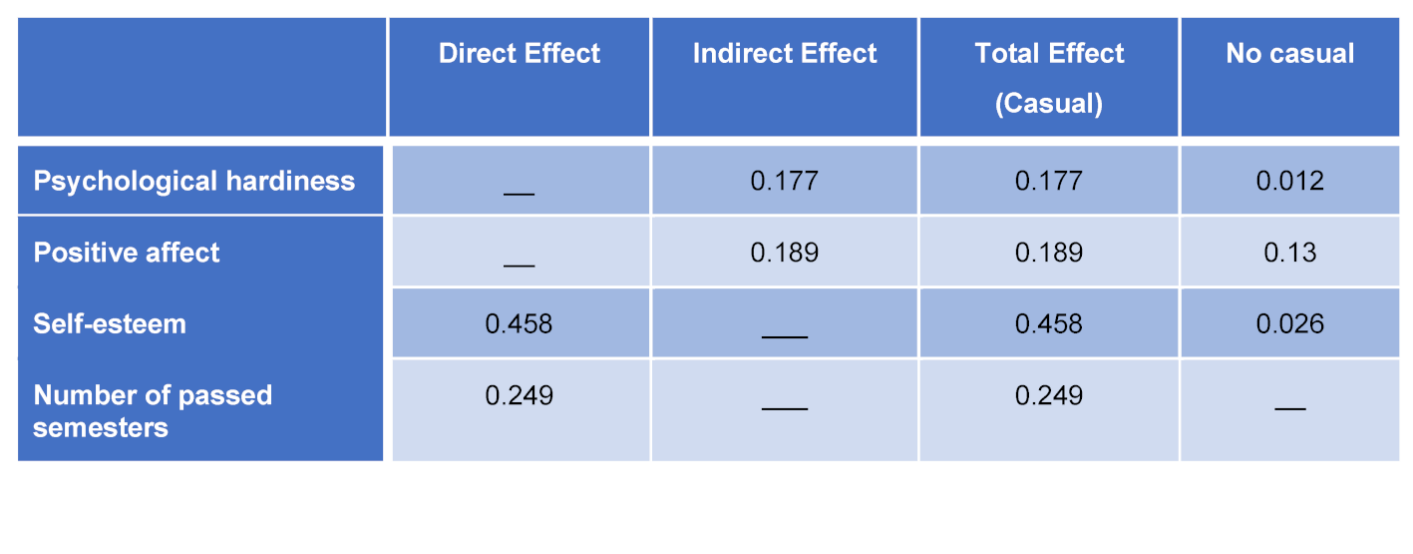
Direct and indirect effect of the psychosocial predictors of critical thinking
